# Wearable Activity Tracker Use and Physical Activity Among Informal Caregivers in the United States: Quantitative Study

**DOI:** 10.2196/40391

**Published:** 2022-11-24

**Authors:** Asos Mahmood, Hyunmin Kim, Satish Kedia, Patrick Dillon

**Affiliations:** 1 Center for Health System Improvement University of Tennessee Health Science Center Memphis, TN United States; 2 Department of Medicine General Internal Medicine University of Tennessee Health Science Center Memphis, TN United States; 3 School of Health Professions The University of Southern Mississippi Hattiesburg, MS United States; 4 School of Public Health The University of Memphis Memphis, TN United States; 5 School of Communication Studies Kent State University North Canton, OH United States

**Keywords:** informal caregivers, caregiving, health and activity trackers, wearables, physical activity, health-promoting behavior, mobile phone

## Abstract

**Background:**

With an increase in aging population and chronic medical conditions in the United States, the role of informal caregivers has become paramount as they engage in the care of their loved ones. Mounting evidence suggests that such responsibilities place substantial burden on informal caregivers and can negatively impact their health. New wearable health and activity trackers (wearables) are increasingly being used to facilitate and monitor healthy behaviors and to improve health outcomes. Although prior studies have examined the efficacy of wearables in improving health and well-being in the general population, little is known about their benefits among informal caregivers.

**Objective:**

This study aimed to examine the association between use of wearables and levels of physical activity (PA) among informal caregivers in the United States.

**Methods:**

We used data from the National Cancer Institute’s Health Information National Trends Survey 5 (cycle 3, 2019 and cycle 4, 2020) for a nationally representative sample of 1273 community-dwelling informal caregivers—aged ≥18 years, 60% (757/1273) female, 75.7% (990/1273) had some college or more in education, and 67.3% (885/1273) had ≥1 chronic medical condition—in the United States. Using jackknife replicate weights, a multivariable logistic regression was fit to assess an independent association between the use of wearables and a binary outcome: meeting or not meeting the current World Health Organization’s recommendation of PA for adults (≥150 minutes of at least moderate-intensity PA per week).

**Results:**

More than one-third (466/1273, 37.8%) of the informal caregivers met the recommendations for adult PA. However, those who reported using wearables (390/1273, 31.7%) had slightly higher odds of meeting PA recommendations (adjusted odds ratios 1.1, 95% CI 1.04-1.77; *P*=.04) compared with those who did not use wearables.

**Conclusions:**

The results demonstrated a positive association between the use of wearables and levels of PA among informal caregivers in the United States. Therefore, efforts to incorporate wearable technology into the development of health-promoting programs or interventions for informal caregivers could potentially improve their health and well-being. However, any such effort should address the disparities in access to innovative digital technologies, including wearables, to promote health equity. Future longitudinal studies are required to further support the current findings of this study.

## Introduction

With a rapidly aging global population, the number of people living with chronic medical conditions (CCs) is increasing. The role of caregivers for this population, including informal caregivers, has become critical as they get involved in the delivery of care and provision of support to patients inside or outside formal health care settings [1]. Evidence suggests that informal caregivers may encounter challenges in providing care, which often requires considerable time and effort. This caregiving burden may result in poorer health outcomes among the caregivers [2,3]. Those caring for patients with Alzheimer disease, other dementias, and cancer are more likely to experience additional burden or distress associated with the caregiving, leading to even worse health outcomes, including depressive symptoms, lower rates of physical inactivity, poorer diet, and insufficient sleep [1,4,5].

Physical activity (PA) is one of the essential components of maintaining good health [6]. PA refers to any body movement or activity generated by skeletal muscles that uses energy, comprising both aerobic exercise and muscle-strengthening activities [7]. The benefits of PA are extensive, including disease prevention, symptom reduction, improved mental health and cognition, and improved quality of life [8-11]. Adults who engage in both aerobic and muscle-strengthening activities, as recommended by national and international PA guidelines, have a 21% to 40% lower risk for all-cause mortality and lower cause-specific cardiovascular mortality [12,13]. Muscle-strengthening activities promote physical and social functioning, reduce body pain, and improve mental and general health status while negating some of effects of CCs and other illnesses [10,11,14]. Despite the importance of PA in maintaining good health [15], a relatively small percentage of people in the United States regularly work out or engage in PA. In 2018, for instance, only 23.2% of the population in the United States met both the recommended levels of aerobic and muscle-strengthening activities [16]. These figures are concerning given that a lack of PA can lead to acute or chronic disease and reduced longevity [17,18]. A systematic review by Reiner et al [18] explored the long-term relationship between PA and selected chronic conditions or diseases (including obesity, type 2 diabetes, Alzheimer disease or dementia, and coronary heart disease) and found that PA appears to be associated with reduced risk for, or preventing, most age-related diseases. On the basis of existing literature, Brown et al [17] examined the effects of PA on healthy brain aging and found that PA can contribute to maintaining improved cognition.

Information and communication technologies—including smartphones and electronic health and activity trackers (henceforth, wearables)—are increasingly being used as tools to facilitate the delivery of care and help improve health outcomes among patients and caregivers. The number of wearable users, as well as willingness to wear these technologies, has substantially grown in recent years both in the United States and worldwide. As of 2022, there are approximately 67 million adult wearable users in the United States, a figure that has increased by almost 42 million users since 2014 [19]. About 21% of adults in the United States (aged ≥18 years) report regularly wearing a smart watch or a wearable fitness tracker, and 53% of adults in the United States show willingness to wear technology that tracks their vital signs and their lifestyle or fitness levels [20,21]. These devices can be used to collect data on PA such as the number of steps taken, calories burned, and heart rate [22,23]. Other sophisticated wearable technology can collect information on blood pressure, glucose levels, blood oxygen saturation, and duration or quality of sleep [23]. Wearables that are embedded in the body or worn as accessories in health care are being increasingly used for monitoring and assessing health [22,24,25]. These tools function by wirelessly sending and receiving various physiological and other health information in an efficient way [26,27]. Mounting evidence suggests the benefits of wearable devices for tracking and monitoring health as safe and cost-effective tools to promote health behavior change such as enhancing PA [28]. Furthermore, the data-generating capabilities of wearables provide substantial value to the users in their health management [29]. In medical settings and patient treatment, incorporating and using wearable data in the health care decision-making process has shown great potential in patient monitoring and enhanced planning and intervention by providing timely feedback [25,29].

Despite numerous studies focused on developing and evaluating wearable devices designed to improve care delivery and health outcomes in the general population [30-32], little is known about how wearable devices impact caregivers, particularly informal caregivers. To our knowledge, no study dealing with this issue has been conducted thus far on a national sample of informal caregivers in the United States. This study examined the association between the use of wearables and PA levels among informal caregivers in the United States. We hypothesize that informal caregivers who use wearables are more likely to be engaged in PA and meet the current World Health Organization (WHO) recommendations of weekly PA of at least moderate intensity compared with informal caregivers who do not use these devices.

## Methods

### Data, Settings, and the Study Sample

For this study, we used data from the Health Information National Trends Survey (HINTS), a nationally representative, cross-sectional, probability-based survey conducted by the United States National Cancer Institute every few years since 2003 [33]. The HINTS, focusing on civilian noninstitutionalized adults aged ≥18 years, compiles information on access to, use of, and needs for health-related information, perceptions, knowledge, and behaviors. However, this survey is not a cancer-specific survey per se [34,35]. More recently, HINTS began collecting information on wearables use as well. We specifically used data from HINTS 5 cycles 3 (2019) and 4 (2020) because these 2 recent cycles of data contain information on wearables use. All 4 cycles of HINTS 5 involved self-administered mailed questionnaires [36] except for cycle 3, which involved a multimode survey that, in addition to the mail-in surveys, incorporated 2 experimental conditions of a web pilot. All mail-in surveys in cycle 3 and all groups in the multimode survey in cycle 4 received a US $2 prepaid monetary incentive to encourage participation; they received an additional US $10 Amazon e-gift card for participating in the second web pilot experimental (Web Bonus) survey in cycle 4.

HINTS 5 cycle 3 data collection began in January 2019 and concluded in April 2019 with an overall response rate of 30.3%. HINTS 5 cycle 4 was fielded between February 2020 and June 2020 with a response rate of 36.7%. The combined HINTS 5 cycles 3 and 4 resulted in an initial unweighted sample of 9303 adults aged ≥18 years. However, our analytical sample included 1273 self-identified informal caregivers. The informal caregiver status was assigned based on participant response to the following two survey questions: “Are you currently caring for or making health care decisions for someone with a medical, behavioral, disability, or other condition?” and “Do you provide any of this care professionally as part of a job (for example, as a nurse or professional home health aide)?”

### PA and Electronic Activity Trackers

The primary outcome of interest in this study was a binary measure indicating whether the informal caregivers were meeting the current WHO recommendations of moderate PA for adults (ie, ≥150 minutes of at least moderate-intensity PA per week) [15]. The measure was derived from a composite of combined participant responses to 2 questions in the HINTS survey. The participants were asked, “In a typical week, how many days do you do any PA or exercise of at least moderate intensity?” They were then asked, “On the days that you do any PA or exercise of at least moderate intensity, how long do you typically do these activities?” On the basis of the participant’ responses, the number of days per week of at least moderate-intensity PA was multiplied by the number of reported minutes per day of PA to compute weekly minutes of at least moderate-intensity PA for each respondent. We classified survey respondents into those with ≥150 minutes of at least moderate-intensity PA per week and those with <150 minutes of at least moderate-intensity PA per week, indicating meeting versus not meeting the current WHO recommendations of moderate PA for adults, respectively. The main independent variable was a binary measure assessing whether the informal caregiver has used wearables during the past 12 months (yes vs no). This indicator was derived from the participant’s response to the survey question, “In the past 12 months, have you used an electronic wearable device to monitor or track your health or activity? For example, a Fitbit, Apple Watch, or Garmin Vivofit*.*”

### Other Explanatory Variables

We followed the constructs of the Social-Ecological Model [37] and Social Cognitive Theory [38] to select study covariates. Drawing from these theoretical frameworks, several sociodemographic characteristics related to informal caregivers were included as control measures in our analyses. Respondents’ ages were categorized into “18 to 34,” “35 to 49,” “50 to 64,” and “≥65” years. Sex was a binary variable, “male versus female.” Each respondent’s race and ethnicity was categorized into “non-Hispanic White,” “non-Hispanic Black,” “Hispanic,” and “non-Hispanic Asian and others.” Marital status was represented through a nominal variable, “married or living as married,” “divorced, widowed, or separated,” and “single or never married.” Informal caregivers’ education was categorized as “less than high school,” “high school graduate,” “some college,” and “college graduate or more.” Other incorporated socioeconomic status characteristics included annual household income, which was categorized as “<US $20,000,” “US $20,000 to US $35,000,” “US $35,001 to US $50,000,” “US $50,001 to US $75,000,” and “>US $75,000.” Other variables included whether the respondents had a smartphone, “yes versus no”; metropolitan statistical area (MSA) residency, “MSA versus non-MSA”; and having a regular provider, “yes versus no.” The incorporated measures of health and health behaviors included number of reported CCs, which was categorized as “none,” “1,” and “≥2.” The number of CCs was constructed based on a history of diagnosed medical conditions including diabetes or high blood sugar; high blood pressure or hypertension; heart conditions such as myocardial infarction, angina, or congestive heart failure; chronic lung disease, asthma, emphysema, or chronic bronchitis; depression or anxiety disorder; and cancer. Smoking status was categorized as “current,” “former,” and “never”; each respondent’s BMI (calculated as weight in kilograms divided by the square of height in meters [kg per m^2^]) was categorized into “underweight or normal (BMI ≤24.9 kg/m^2^),” “overweight (BMI=25-29.9 kg/m^2^),” and “obese (BMI ≥30kg/m^2^).” We also incorporated 2 measures of caregiver self-efficacy: self-rated general health status—which was categorized as “excellent or very good,” “good,” and “fair or poor”—and caregiver’s self-reported confidence in taking care of own health—categorized as “completely confident,” “very confident,” and “somewhat, a little, or not confident at all.”

### Statistical Approach

We first calculated the unweighted frequencies and weighted proportions for the entire sample of informal caregivers and then by subgroups based on PA levels. Wald chi-square was used to test for equal proportions in 2-way analyses. Univariate and multivariable logistic regressions were fit to assess the association between the use of wearables and the binary PA outcome. The fully adjusted model incorporated the primary independent variable and the entire pool of selected covariates. Multicollinearities were checked, and the significance of interaction terms was assessed by the likelihood ratio test. Assessing for multicollinearities was performed by first exploring the correlation matrix and then the variance inflation factor and tolerance. There were no threats of multicollinearity between the model variables. The final generated outputs included odds ratios (ORs), their 95% CIs, and associated *P* values. In the above analytical steps, final person weights and jackknife replicate weights from the HINTS data set were used to estimate national-level values and more accurate SEs of estimates. The significance threshold was set at *P*<.05. All analyses were performed using the SAS statistical software (version SAS 9.4; SAS Institute Inc).

### Ethical Considerations

This study involved analyses of secondary data from the HINTS 5 data set, which is primarily deidentified and publicly available. The institutional review board of Westat, the organization that administers the survey, and the institutional review board of the National Cancer Institute Office of Human Subjects Research both granted exempted status for the use and analysis of HINTS data. Additional details about the HINTS survey design, methodology, and access to public data can be found on the survey website [39].

## Results

The analytical sample of 1273 caregivers represented a national-level estimate of approximately 73.1 million informal caregivers in the United States. Table 1 shows unweighted sample frequencies and weighted national-level proportions for characteristics of the informal caregivers. Approximately 37.8% (466/1273) of caregivers reported ≥150 minutes of at least moderate-intensity PA per week, whereas about one-third (390/1273, 31.7%) of them reported using wearables during the past 12 months. About 56.3% (813/1273) of caregivers were aged ≥50 years, 60% (757/1273) were females, and 63.6% (706/1273) were non-Hispanic White adults. Other characteristics included the following: 70% (815/1273) were married or living as married, 75.7% (990/1273) had some college or more in education, 44.5% (491/1273) had an annual household income of >US $75,000, 89.7% (1058/1273) had a smartphone, 88.1% (1144/1273) were residing in an MSA in the United States, and 72% (935/1273) reported having a regular provider. A large proportion of caregivers (885/1273, 67.3%) had ≥1 CC, 38.3% (461/1273) were current or former smokers, and 38% (477/1273) were obese (BMI of ≥30 kg/m^2^). Of the 1273 informal caregivers, 578 (44.2%) rated their general health status as excellent or very good, and 67.9% (904/1273) of the informal caregivers were completely confident or very confident about taking care of their own health (Table 1).

Among those meeting the recommendations of engaging in ≥150 minutes of at least moderate-intensity PA per week, approximately 43.1% (201/466) reported using wearables during the past 12 months (Table 1). For this specific subgroup of caregivers, 54.4% (283/466) were aged ≥50 years, 54.7% (252/466) were females, and 60.9% (278/466) were non-Hispanic White adults. Furthermore, almost 75% (315/466) of the caregivers were married or living as married, 84.1% (583/466) had some college or more in education, 55.3% (224/466) had an annual household income >US $75,000, 92.2% (411/466) had a smartphone, 89.6% (424/466) were residing in an MSA in the United States, and 71.7% (345/466) had a regular health care provider. About 62.4% (293/466) of the caregivers had ≥1 CC, 33.4% (153/466) were current or former smokers, and 37.3% (176/466) were either under or normal weight (BMI ≤24.9 kg/m^2^). Approximately 56.6% (278/466) of the caregivers reported their general health status as being excellent or very good, and 77.9% (376/466) of the caregivers reported that they were completely confident or very confident about taking care of their own health.

From our multivariable logistic regression model, informal caregivers who reported wearable use during the past 12 months had higher odds (adjusted OR 1.1, 95% CI 1.04-1.77; *P*=.04) of engaging in ≥150 minutes of at least moderate-intensity PA per week compared with those who did not use wearables (Table 2). Apart from wearables use, caregiver’s annual household income and self-rated general health status were associated with the levels of PA. Caregivers with an income of US $20,000 to US $35,000 (adjusted OR 2.67, 95% CI 1.01-7.08; *P*=.048), US $35,001 to US $50,000 (adjusted OR 2.91, 95% CI 1.17-7.24; *P*=.02), US $50,001 to US $75,000 (adjusted OR 3.72, 95% CI 1.28-10.81; *P*=.02), and >US $75,000 (adjusted OR 3.70, 95% CI 1.51-9.60; *P*=.005) had higher odds of engaging in ≥150 minutes of at least moderate-intensity PA per week relative to caregivers with an annual income of <US $20,000. Caregivers who self-rated their health as fair or poor had lower odds (adjusted OR 0.39, 95% CI 0.16-0.94; *P*=.04) of engaging in ≥150 minutes of at least moderate-intensity PA per week compared with caregivers who rated their own health as excellent or very good.

**Table 1:** Informal caregiver characteristics in the United States (HINTS^a^ 5—cycles 3, 2019 and 4, 2020; N=1273).

Characteristics		Sample, frequency (weighted %)^b^	Minutes per week of at least moderate-intensity exercise, frequency (weighted %)^b^		*P* values opted from Wald *χ*^2^ test
			≥150 minutes (n=466)	<150 minutes (n=764)	
**Minutes per week of at least moderate-intensity exercise**					
	≥150 minutes	466 (37.8)	—^c^	—	—
	<150 minutes	764 (62.2)	—	—	—
**Electronic wearable device use^d^**					
	Yes	390 (31.7)	201 (42.1)	179 (26.4)	.03
	No	873 (68.3)	262 (57.9)	581 (73.6)	—
**Age groups (years)**					
	18-34	103 (11.7)	35 (9.9)	67 (13.1)	.62
	35-49	318 (32)	136 (35.7)	180 (30.5)	—
	50-64	457 (39.6)	166 (39.1)	284 (40.7)	—
	≥65	356 (16.7)	117 (15.3)	218 (15.7)	—
**Sex**					
	Male	426 (40)	184 (45.3)	232 (37.4)	.06
	Female	757 (60)	252 (54.7)	488 (62.6)	—
**Race and ethnicity**					
	Hispanic	188 (15.3)	63 (15.8)	119 (14.7)	—
	Non-Hispanic Asian and others	104 (12)	38 (14.0)	66 (11.1)	—
	Non-Hispanic Black	150 (9.1)	54 (9.3)	93 (9.1)	—
	Non-Hispanic White	706 (63.6)	278 (60.9)	410 (65.1)	.86
**Marital status**					
	Married or living as married	815 (70)	315 (74.8)	484 (67.5)	.13
	Divorced, widowed, or separated	268 (12.4)	85 (10.2)	172 (13.3)	—
	Single or never married	149 (17.6)	55 (15)	91 (19.2)	—
**Education**					
	Less than high school	66 (5.2)	21 (5.4)	40 (4.5)	.002
	High school graduate	182 (19.1)	45 (10.5)	128 (23.7)	—
	Some college	372 (43.8)	138 (45.4)	226 (43.2)	—
	College graduate or more	618 (31.9)	243 (38.7)	357 (28.6)	—
**Annual household income (US $)**					
	<20,000	184 (16.3)	47 (8.3)	124 (19.6)	.004
	20,000-35,000	121 (10.9)	37 (9.2)	81 (11.8)	—
	35,001-50,000	138 (11.2)	51 (9.6)	84 (12.3)	—
	50,001-75,000	210 (17.1)	69 (17.6)	137 (16.9)	—
	>75,000	491 (44.5)	224 (55.3)	262 (39.4)	—
**Have a smartphone^e^**					
	Yes	1058 (89.7)	411 (92.2)	624 (88.7)	.17
	No	188 (10.3)	45 (7.8)	128 (11.3)	—
**MSA^f^ residency**					
	MSA	1144 (88.1)	424 (89.6)	682 (86.9)	.46
	Non-MSA	129 (11.9)	42 (10.4)	82 (13.1)	—
**Have a regular provider**					
	Yes	935 (72)	345 (71.7)	570 (73.0)	.77
	No	323 (28)	116 (28.3)	190 (27)	—
**Chronic medical conditions**					
	None	384 (32.7)	173 (37.6)	200 (30.2)	.09
	1	393 (31.6)	146 (32.4)	235 (31)	—
	≥2	492 (35.7)	147 (30)	328 (38.8)	—
**Smoking status**					
	Current	138 (13.2)	54 (14.4)	82 (12.3)	.04
	Former	323 (25.1)	99 (19)	214 (28.1)	—
	Never	790 (61.7)	310 (66.6)	461 (59.6)	—
**BMI (kg/m^2^)**					
	Underweight or normal (≤24.9)	379 (31.6)	176 (37.3)	187 (27.5)	.02
	Overweight (25-29.9)	385 (30.4)	155 (31.7)	222 (29.5)	—
	Obese (≥30)	477 (38)	127 (31)	339 (43)	—
**Self-rated general health status**					
	Excellent or very good	578 (44.2)	278 (56.6)	283 (37)	<.001
	Good	478 (38.6)	145 (33.5)	320 (41.7)	—
	Fair or poor	207 (17.2)	41 (9.9)	156 (21.3)	—
**Confidence in taking care of own health**					
	Completely confident	304 (23.6)	153 (31.2)	138 (19)	<.001
	Very confident	600 (44.3)	223 (46.7)	362 (42.9)	—
	Somewhat, a little, or not confident at all	367 (32.1)	90 (22.1)	264 (38.1)	—

^a^HINTS: Health Information National Trends Survey.

^b^Frequencies represent sample frequencies; proportions are population-level estimates that were generated by adjusting for complex survey features of the HINTS data (N=73.1 million).

^c^Not available.

^d^Such as Fitbit, AppleWatch, or Garmin Vivofit.

^e^Such as iPhone, Android, Blackberry, or Windows phone.

^f^MSA: metropolitan statistical area.

**Table 2:** Logistic regressions modeling the association between use of electronic activity trackers (wearables) and meeting recommendations of physical activity (≥150 minutes per week of at least moderate-intensity exercise) among informal caregivers.

Characteristics			Crude OR^a^ (95% CI)		Adjusted OR (95% CI)
**Electronic wearable device use^b^**					
	Yes	1.9 (1.12-2.26)^c^		1.1 (1.04-1.77)^c^	
	No	Reference		Reference	
**Age groups (years)**					
	18-34	Reference		Reference	
	35-49	1.55 (0.77-3.13)		1.06 (0.46-2.45)	
	50-64	1.27 (0.67-2.44)		0.79 (0.38-1.64)	
	≥65	1.3 (0.64-2.64)		0.98 (0.38-2.51)	
**Sex**					
	Male	Reference		Reference	
	Female	0.72 (0.51-1.02)		0.72 (0.43-1.22)	
**Race and ethnicity**					
	Hispanic	1.15 (0.67-1.96)		1.5 (0.79-2.85)	
	Non-Hispanic Asian and others	1.35 (0.61-2.99)		1.39 (0.49-3.99)	
	Non-Hispanic Black	1.09 (0.61-1.94)		1.85 (0.77-4.43)	
	Non-Hispanic White	Reference		Reference	
**Marital status**					
	Married or living as married	1.42 (0.82-2.48)		1.08 (0.53-2.21)	
	Divorced, widowed, or separated	0.98 (0.52-1.88)		1.19 (0.45-3.13)	
	Single or never married	Reference		Reference	
**Education**					
	Less than high school	Reference		Reference	
	High school graduate	0.36 (0.14-0.94)^c^		0.32 (0.1-1.26)	
	Some college	0.86 (0.33-2.25)		0.55 (0.15-1.99)	
	College graduate or more	1.11 (0.46-2.69)		0.57 (0.15-2.13)	
**Annual household income** **(US $)**					
	<20,000	Reference		Reference	
	20,000-35,000	1.86 (0.95-3.67)		2.67 (1.01-7.08)^c^	
	35,001-50,000	1.86 (0.94-3.65)		2.91 (1.17-7.24)^c^	
	50,001-75,000	2.46 (1.21-4.99)^c^		3.72 (1.28-10.81)^c^	
	>75,000	3.32 (1.85-5.95)^d^		3.8 (1.51-9.6)^e^	
**Have a smartphone^f^**					
	Yes	1.52 (0.81-2.85)		0.82 (0.31-2.13)	
	No	Reference		Reference	
**MSA^g^ residency**					
	MSA	Reference		Reference	
	Non-MSA	0.77 (0.36-1.64)		1.3 (0.54-3.16)	
**Have a regular provider**					
	Yes	0.94 (0.6-1.46)		1.13 (0.61-2.08)	
	No	Reference		Reference	
**Chronic medical conditions**					
	None	Reference		Reference	
	1	0.84 (0.53-1.32)		1.08 (0.61-1.92)	
	≥2	0.62 (0.41-0.95)^c^		1.19 (0.66-2.15)	
**Smoking status**					
	Current	Reference		Reference	
	Former	0.58 (0.28-1.2)		0.47 (0.19-1.13)	
	Never	0.96 (0.49-1.88)		0.73 (0.31-1.68)	
**BMI (kg/m^2^)**					
	Underweight or normal (≤24.9)	Reference		Reference	
	Overweight (25-29.9)	0.79 (0.49-1.29)		0.90 (0.47-1.69)	
	Obese (≥30)	0.53 (0.33-0.85)^e^		0.62 (0.31-1.23)	
**Self-rated general health status**					
	Excellent or very good	Reference		Reference	
	Good	0.53 (0.38-0.73)^d^		0.66 (0.38-1.13)	
	Fair or poor	0.30 (0.16-0.57)^d^		0.39 (0.16-0.94)^c^	
**Confidence in taking care of own health**					
	Completely confident	Reference		Reference	
	Very confident	0.66 (0.43-1.02)		0.81 (0.45-1.47)	
	Somewhat, a little, or not confident at all	0.35 (0.22-0.56)^d^		0.74 (0.37-1.46)	

^a^OR: odds ratio.

^b^Such as Fitbit, AppleWatch, or Garmin Vivofit.

^c^*P*<.05.

^d^*P*<.001.

^e^*P*<.01.

^f^Such as iPhone, Android, Blackberry, or Windows phone.

^g^MSA: metropolitan statistical area.

## Discussion

### Principal Findings and Implications for Policy and Practice

Using a nationally representative sample of 1273 informal caregivers in the United States (73.1 million at the national level), we examined whether informal caregivers who use wearables met the current WHO recommendations of ≥150 minutes of at least moderate-intensity PA for adults. Study findings revealed that informal caregivers who reported using wearables during the past 12 months had higher odds of meeting PA recommendations. Informal caregivers are pillars of current health care systems, as they provide essential care and emotional support to their loved ones. However, they often experience significant burden associated with their caregiving roles, which can negatively impact their health and well-being. Those who care for older patients with CCs (ie, cancer, hypertension, etc), dementia or Alzheimer disease are even more likely to report poorer physical and mental health as well as social and financial challenges attributed to the caregiving burden, including limited time and resources.

Mounting evidence exists about the use of information technologies and their health-related benefits among various groups of populations, including informal caregivers [30-32]. For instance, Matthews et al [32] assessed how family caregivers deal with challenging aspects of dementia to inform formal interventions designed to strengthen their caregiving knowledge and skills. The findings indicated that family caregivers of people with dementia could use a novel, wearable camera system to collect evidence of dementia-related behaviors and interactions that may affect the health and safety of the caregiving dyad. Furthermore, Egan et al [30] co-designed and assessed a mobile app named CareFit to educate and support caregivers to perform regular PA at home during and after COVID-19 restrictions by integrating a transtheoretical model of behavior change based on the United Kingdom’s guidelines for PA. They found that integrating PA into the CareFit app with functions such as a weekly planner and educational material for users is feasible. In addition, an observational study by Martinato et al [31] assessed Vivoactive HR (Garmin) smartwatch as a wearable device in quantifying PA in a sample of 49 older adults and found the potential of the wearable device to enhance PA among care recipients by capturing even the low levels of PA. Furthermore, Jaschinski and Allouch [40] found that most informal caregivers had a positive perception about using ambient assisted living and appreciated the help of ambient assisted living technologies in preventing accidents and alerting them immediately in case of an emergency [40].

There is a greater potential for wearable devices to improve health and health care. Wearable technology can serve as a safe and cost-effective intervention to promote health and healthy behaviors such as PA [28]. Incorporating health-related data obtained from wearables into the electronic health record systems could further assist health care professionals in monitoring individual health status and providing relevant care and support efficiently [25,28,41]. Notably, by connecting or linking to mobile apps, wearables can better facilitate motivating and managing individual health and health care [42]. Furthermore, prior systematic reviews and meta-analysis in this domain have provided evidence regarding the positive influence of using wearable technology in increasing PA among various subgroups of population [43-45]. The use of wearable devices as a health-promoting intervention is associated with increased PA and steps per day and a significant increase in moderate to vigorous minutes per week of PA among patients with cardiometabolic diseases [44]. Other studies indicate that using consumer-based wearable activity trackers as an intervention modality significantly increased daily step count and energy expenditure among wearable users compared with those who do not use these devices among a wide range of healthy populations and populations with CC [43].

Nonetheless, there is the issue of the digital divide related to these innovative technologies, which could worsen the already existing disparities [2,46]. The digital divide pertains to differences between have’s and have-not’s with information and communication technologies [47]. The gap in access to digital technologies, including wearable devices, can widen the already present health and health care disparities [2]. Evidence shows that the younger, more educated, wealthier, and tech-savvy adults are more likely to use wearables compared with older adults, those with lower education and income, and non–tech-savvy individuals [21,28,48]. Farivar et al [49] explored the extent to which factors were associated with the adoption of wearable devices among older adults by conducting a mixed methods study and found their perceived complexity in using wearable devices as a barrier to adoption. In addition, it was found that cognitive age itself does not considerably affect wearable device use intention, which is moderated by subjective well-being in older adults, indicating that older adults’ use intention is influenced by their subjective well-being [49].

Our findings show that informal caregivers with higher income, compared with those with lower income (≤US $20,000), were more likely to engage in PA by meeting the recommended guidelines. A meta-analysis conducted by Pinquart and Sörensen [50] examined correlates of informal caregivers’ physical health and found that having higher income was associated with better physical health. They suggested that less access to health care and inadequate health practices may be attributed to the relationship of income with physical health [51]. As one of the socioeconomic indicators, income is considered a correlate of financial resources and tangible well-being that may affect individual’s healthy behaviors [51]. Insufficient tangible resources may influence individual health behaviors from financial limitations that can hinder people from making healthy choices, albeit not all those choices are based on income or money [51]. Nonetheless, evidence also suggests that the relation of socioeconomic components, including income, to individual health behaviors is not clear or straightforward [51]. Moreover, other evidence from the United States indicates that use of wearable devices and other digital technologies is greatly impacted by individuals’ socioeconomic status [21]. Approximately 31% of the residents of the United States with an annual household income of ≥US $75,000 reported wearing a smart watch or fitness tracker on a regular basis, whereas only 12% of those with an annual household income ≤US $30,000 reported using these devices [21]. Similarly, people with higher educational attainment are more likely to adopt these technologies than those with lower levels of education [21].

Interestingly, our findings also indicated that informal caregivers who assessed their health as fair or poor, when compared with those who rated their health as excellent or very good, were less likely to meet PA recommendations. This finding was in line with that of prior studies that reported positive associations of higher self-efficacy and self-rated health with initiation of exercise, higher PA, and better health-promoting behaviors [52-55]. Thus, these psychosocial factors are important predictors of PA among informal caregivers. Specifically, lower levels of perceived health may actually be a potential barrier to PA [54]. It is also possible that this group of informal caregivers may not have had sufficient time to engage in PA owing to their caregiving burden. Indeed, informal caregivers have challenging circumstances or situations such as financial difficulties, time constraints, and other barriers related to their caregiving role [2]. In this respect, innovative technologies, including wearables, could help reduce the burden associated with caregiving by providing beneficial features (eg, motivation, self-tracking, information gathering and exchange, and communication with a provider), which could potentially help improve individual health and well-being [2]. Notably, they could be used by individuals to further engage them in health-related activities, including PA [30], particularly caregivers who are more likely to have poorer physical and mental health and emotional well-being.

There is a greater need for providing support and help to informal caregivers, given that they play an essential role in the health care system. Incorporating elements of innovative information and communication technologies such as wearables could contribute to beneficial health outcomes. Wearables can help individuals be proactive in monitoring, tracking, and managing their health and health care and can help improve their quality of life [56]. Wearables are becoming more affordable and easier to use for monitoring health-related physical activities or conditions, particularly among older people. Wearable devices are beneficial to overcome the diverse needs related to aging among older adults [57]. Essentially, health-promoting strategies, such as providing health education materials or counseling, should be provided using these technologies to promote or enhance health and health care [23,58]. To reduce the burden associated with caregiving, training should be offered to them, especially those who are new to caregiving. For example, caregiver stress and burden could be reduced by providing instructions about how to perform tasks such as offering mobility assistance, advocating for medical treatment, administering medications and injections, and using digital technologies and other electronic tools (eg, personal health record tracking and medication support systems) [59,60].

Given that most caregivers have a smartphone, they can download and use mobile health apps for health-related purposes. For example, downloading a pedometer app could help enhance their PA. Moreover, facilitating the use of wearables and making them less noticeable and user-friendly, while providing appropriate operating instructions, could help reduce anxiety about technology use [61,62]. Today, there are different types of products (smartwatches, fitness trackers, smart clothes, implantable gadgets, head-mounted displays, etc) from various manufacturers (Xiaomi, Huawei, Polar, Samsung, Apple, Garmin Ltd, Withings, and Fitbit just to name a few) that are available in the market; the variety of options can be daunting for informal caregivers when making decisions about which wearable device to adopt and use [62,63].

### Limitations

Although this study provides novel insights that have implications for health care policy and practice, it has several limitations. First, owing to the cross-sectional nature of the HINTS survey, we were unable to infer causality in the reported associations. Second, despite capturing the relevant variables based on the conceptual and theoretical frameworks, it is possible that there could be a more appropriate conceptual model. However, given that the use and implementation of wearable devices are still developing and in their infancy, unified and better-fitting theories and frameworks related to this field are still being developed [64]. Third, provided that the data used for this study were based on a national survey including the self-reported information, there could be recall bias if the respondents did not provide correct information, and there is a possibility that other types of bias may be introduced, including social desirability bias. Fourth, despite ongoing efforts to improve the HINTS survey response rates [65], these rates have remained low, and there is potential for selection bias; thus, population representativeness and generalizability of the findings could be questionable. Fifth, although we adjusted for several related factors based on the theoretical frameworks and literature review, there may be other factors that were not included in our model. For example, we could not adjust for many contextual factors or attributes possibly associated with the use of wearables or PA. Sixth, no thorough assessment of muscle-strengthening activities was provided in the database, which could have supplemented our evaluation of PA. Finally, other critical information about consistency of wearables use, device features, and quality of engagement of those who used the device during the past 12 months were also not collected in this survey. Thus, we were constrained in our analyses by the information provided in the HINTS data set. All these limitations present an opportunity for our team and other researchers to expand the study in the future. Potentially more prospective studies can be designed to further support the findings of this study. In addition, investigating these associations among groups of caregivers who specifically care for patients with CCs (eg, diabetes, hypertension, cancer, and dementia or Alzheimer disease) will be of particular interest in the caregiving field.

### Conclusions

In this study, we used a nationally representative sample of 1273 self-identified informal caregivers in the United States to assess the associations between use of wearables and the status of meeting the current WHO recommendations of moderate-intensity PA for adults (ie, ≥150 minutes of at least moderate-intensity PA per week). We found that informal caregivers who reported wearable use during the past 12 months were modestly more likely to engage in ≥150 minutes of at least moderate-intensity PA per week compared with caregivers who did not use wearables. The results demonstrated the potentials of wearables as a means of increasing PA among informal caregivers, thus their role in health promotion and improving quality of life among this important segment of the population.

Long-term adoption could potentially be critical for the delivery of the benefits promised by wearable technology, and yet, this particular area of research requires further scrutiny [64]. There are several acceptance- and abandonment-related issues that substantially influence long-term wearable use. Some of these factors include device appearance, display and interaction, wearability, perceived usefulness or risks, and other technical issues such as data measurement and presentation [29,64]. Other factors that influence wearable use include development of more personalized devices for various groups of people who have different needs and preferences. Usually, designing of an all-purpose wearable is unreasonable and less impactful and is less likely to have sustained use or benefits over time [29].

There are many other issues related to wearable development and use that need to be properly addressed. A few of these issues include data security and protection, consumer privacy concerns, device accuracy, discoverability risks, ethical issues related to the tracking features of wearables, and the fact that many wearables are not regulated by the United States Food and Drug Administration [22,23,25,29,66]. Furthermore, other issues related to wearable technology adoption such as lack of awareness regarding the health and well-being benefits of wearable device use and their implications for physical and mental health, specifically among caregiving and older adults, need to be particularly addressed [23]. To overcome these challenges and concerns, wearable technology developers, researchers, interventionists, health care providers, and policy makers should work in synergy to design and develop personalized, effective, and validated wearables with an optimum impact on health behavior change and health promotion for the caregiver population [25]. The role of digital divide in the adoption and effectiveness of wearables should be emphasized and addressed [64]. As most health technology adoptions are lagging among those in low-income and low-education subgroups, the design and development of wearables should overcome the initial and long-term barriers of adoption among this disadvantaged segment of the population.

## Abbreviations

CC: chronic medical condition
HINTS: Health Information National Trends Survey
MSA: metropolitan statistical area
OR: odds ratio
PA: physical activity
WHO: World Health Organization

## Data Availability

The data analyzed for this study were obtained from the National Cancer Institute’s Health Information National Trends Survey; it is publicly available for access and download at https://hints.cancer.gov/.

## References

[1] Wittenberg Y, Kwekkeboom R, Staaks J, Verhoeff A, de Boer A (2018). Informal caregivers' views on the division of responsibilities between themselves and professionals: a scoping review. Health Soc Care Community.

[2] Kim H, Mahmood A, Goldsmith JV, Chang H, Kedia S, Chang CF (2021). Access to broadband internet and its utilization for health information seeking and health communication among informal caregivers in the United States. J Med Syst.

[3] Tonsaker T, Law S, Ormel I, Nease C, Bartlett G (2017). Engaging caregivers: exploring perspectives on web-based health information. Fam Pract.

[4] Halpern M, Fiero M, Bell M (2017). Impact of caregiver activities and social supports on multidimensional caregiver burden: analyses from nationally-representative surveys of cancer patients and their caregivers. Qual Life Res.

[5] Kim H, Goldsmith J, Sengupta S, Mahmood A, Powell M, Bhatt J, Chang C, Bhuyan S (2019). Mobile health application and e-health literacy: opportunities and concerns for cancer patients and caregivers. J Cancer Educ.

[6] Rhodes RE, Janssen I, Bredin SS, Warburton DE, Bauman A (2017). Physical activity: health impact, prevalence, correlates and interventions. Psychol Health.

[7] (2022). Physical activity. World Health Organization.

[8] Fox KR (2007). The influence of physical activity on mental well-being. Public Health Nutr.

[9] Teychenne M, White RL, Richards J, Schuch FB, Rosenbaum S, Bennie JA (2020). Do we need physical activity guidelines for mental health: what does the evidence tell us?. Mental Health Physical Activity.

[10] Khodadad Kashi S, Mirzazadeh Z, Saatchian V (2022). A systematic review and meta-analysis of resistance training on quality of life, depression, muscle strength, and functional exercise capacity in older adults aged 60 years or more. Biol Res Nurs.

[11] Mahmoudi A, Amirshaghaghi F, Aminzadeh R, Mohamadi Turkmani E (2022). Effect of aerobic, resistance, and combined exercise training on depressive symptoms, quality of life, and muscle strength in healthy older adults: a systematic review and meta-analysis of randomized controlled trials. Biol Res Nurs.

[12] Zhao M, Veeranki S, Magnussen C, Xi B (2020). Recommended physical activity and all cause and cause specific mortality in US adults: prospective cohort study. BMJ.

[13] Saeidifard F, Medina-Inojosa J, West C, Olson T, Somers V, Bonikowske A, Prokop L, Vinciguerra M, Lopez-Jimenez F (2019). The association of resistance training with mortality: a systematic review and meta-analysis. Eur J Prev Cardiol.

[14] Ross LM, Porter RR, Durstine JL (2016). High-intensity interval training (HIIT) for patients with chronic diseases. J Sport Health Sci.

[15] Bull FC, Al-Ansari SS, Biddle S, Borodulin K, Buman MP, Cardon G, Carty C, Chaput J, Chastin S, Chou R, Dempsey PC, DiPietro L, Ekelund U, Firth J, Friedenreich CM, Garcia L, Gichu M, Jago R, Katzmarzyk PT, Lambert E, Leitzmann M, Milton K, Ortega FB, Ranasinghe C, Stamatakis E, Tiedemann A, Troiano RP, van der Ploeg HP, Wari V, Willumsen JF (2020). World Health Organization 2020 guidelines on physical activity and sedentary behaviour. Br J Sports Med.

[16] (2022). Exercise or physical activity. Centers for Disease Control and Prevention.

[17] Brown B, Peiffer J, Taddei K, Lui J, Laws S, Gupta V, Taddei T, Ward V, Rodrigues M, Burnham S, Rainey-Smith S R, Villemagne V L, Bush A, Ellis K A, Masters C L, Ames D, Macaulay S L, Szoeke C, Rowe C C, Martins R N (2013). Physical activity and amyloid-β plasma and brain levels: results from the Australian Imaging, Biomarkers and Lifestyle Study of Ageing. Mol Psychiatry.

[18] Reiner M, Niermann C, Jekauc D, Woll A (2013). Long-term health benefits of physical activity--a systematic review of longitudinal studies. BMC Public Health.

[19] Laricchia F (2022). Number of wearable device users in the United States from 2014 to 2022. Statista.

[20] Stewart C (2019). Percentage of U.S. adults that were willing to wear technology that tracks select health statistics as of 2018. Statista.

[21] Vogels EA (2020). About one-in-five Americans use a smart watch or fitness tracker. Pew Research Center.

[22] Edington DW, Schultz AB, Pitts JS, Camilleri A (2016). The future of health promotion in the 21st century: a focus on the working population. Am J Lifestyle Med.

[23] Wu M (2019). Wearable technology applications in healthcare: a literature review. Online J Nurs Inf.

[24] Fraile JA, Bajo J, Corchado JM, Abraham A (2010). Applying wearable solutions in dependent environments. IEEE Trans Inform Technol Biomed.

[25] Kang H, Mahoney D, Hoenig H, Hirth V, Bonato P, Hajjar I, Lipsitz L, Center for Integration of MedicineInnovative Technology Working Group on Advanced Approaches to Physiologic Monitoring for the Aged (2010). In situ monitoring of health in older adults: technologies and issues. J Am Geriatr Soc.

[26] Baig MM, Afifi S, GholamHosseini H, Mirza F (2019). A systematic review of wearable sensors and IoT-based monitoring applications for older adults - a focus on ageing population and independent living. J Med Syst.

[27] Vollmer Dahlke D, Lee S, Smith ML, Shubert T, Popovich S, Ory MG (2021). Attitudes toward technology and use of fall alert wearables in caregiving: survey study. JMIR Aging.

[28] Xie Z, Jo A, Hong Y (2020). Electronic wearable device and physical activity among US adults: an analysis of 2019 HINTS data. Int J Med Inform.

[29] Shin G, Jarrahi MH, Fei Y, Karami A, Gafinowitz N, Byun A, Lu X (2019). Wearable activity trackers, accuracy, adoption, acceptance and health impact: a systematic literature review. J Biomed Inform.

[30] Egan KJ, Clark P, Deen Z, Paputa Dutu C, Wilson G, McCann L, Lennon M, Maguire R (2022). Understanding current needs and future expectations of informal caregivers for technology to support health and well-being: national survey study. JMIR Aging.

[31] Martinato M, Lorenzoni G, Zanchi T, Bergamin A, Buratin A, Azzolina D, Gregori D (2021). Usability and accuracy of a smartwatch for the assessment of physical activity in the elderly population: observational study. JMIR Mhealth Uhealth.

[32] Matthews JT, Lingler JH, Campbell GB, Hunsaker AE, Hu L, Pires BR, Hebert M, Schulz R (2015). Usability of a wearable camera system for dementia family caregivers. J Healthc Eng.

[33] (2017). Health Information National Trends Survey 5 (HINTS 5) Cycle 1 Methodology Report. National Cancer Institute.

[34] Hesse BW, Moser RP, Rutten LJ, Kreps GL (2006). The health information national trends survey: research from the baseline. J Health Commun.

[35] Nelson D, Kreps G, Hesse B, Croyle R, Willis G, Arora N, Rimer B, Viswanath K V, Weinstein N, Alden S (2004). The Health Information National Trends Survey (HINTS): development, design, and dissemination. J Health Commun.

[36] About HINTS. National Cancer Institute.

[37] Bronfenbrenner U (1977). Toward an experimental ecology of human development. Am Psychologist.

[38] Bandura A (2002). Social foundations of thought and action. The Health Psychology Reader.

[39] What is HINTS?. National Cancer Institute.

[40] Jaschinski C, Ben Allouch S (2018). Listening to the ones who care: exploring the perceptions of informal caregivers towards ambient assisted living applications. J Ambient Intell Human Comput.

[41] Dinh-Le C, Chuang R, Chokshi S, Mann D (2019). Wearable health technology and electronic health record integration: scoping review and future directions. JMIR Mhealth Uhealth.

[42] Lyons EJ, Lewis ZH, Mayrsohn BG, Rowland JL (2014). Behavior change techniques implemented in electronic lifestyle activity monitors: a systematic content analysis. J Med Internet Res.

[43] Brickwood K, Watson G, O'Brien J, Williams AD (2019). Consumer-based wearable activity trackers increase physical activity participation: systematic review and meta-analysis. JMIR Mhealth Uhealth.

[44] Kirk MA, Amiri M, Pirbaglou M, Ritvo P (2019). Wearable technology and physical activity behavior change in adults with chronic cardiometabolic disease: a systematic review and meta-analysis. Am J Health Promot.

[45] Wang JB, Cataldo JK, Ayala GX, Natarajan L, Cadmus-Bertram LA, White MM, Madanat H, Nichols JF, Pierce JP (2016). Mobile and wearable device features that matter in promoting physical activity. J Mob Technol Med.

[46] Mahmood A, Kedia S, Wyant D, Ahn S, Bhuyan S (2019). Use of mobile health applications for health-promoting behavior among individuals with chronic medical conditions. Digit Health.

[47] Talukdar D, Gauri DK (2011). Home internet access and usage in the USA: trends in the socio-economic digital divide. Commun Assoc Inf Syst.

[48] Wójcik D, Szczechowiak K, Konopka P, Owczarek M, Kuzia A, Rydlewska-Liszkowska I, Pikala M (2021). Informal dementia caregivers: current technology use and acceptance of technology in care. Int J Environ Res Public Health.

[49] Farivar S, Abouzahra M, Ghasemaghaei M (2020). Wearable device adoption among older adults: a mixed-methods study. Int J Inf Manage.

[50] Pinquart M, Sorensen S (2007). Correlates of physical health of informal caregivers: a meta-analysis. J Gerontology Series B Psychological Sci Social Sci.

[51] Laaksonen M, Prättälä R, Helasoja V, Uutela A, Lahelma E (2003). Income and health behaviours. Evidence from monitoring surveys among Finnish adults. J Epidemiol Community Health.

[52] Pan SY, Cameron C, Desmeules M, Morrison H, Craig CL, Jiang X (2009). Individual, social, environmental, and physical environmental correlates with physical activity among Canadians: a cross-sectional study. BMC Public Health.

[53] Weiss DR, O'Loughlin JL, Platt RW, Paradis G (2007). Erratum To: five-year predictors of physical activity decline among adults in low-income communities: a prospective study. Int J Behav Nutr Phys Act.

[54] Towers A, Flett R, Seebeck R (2005). Assessing potential barriers to exercise adoption in middle-aged men: over-stressed, under-controlled, or just too unwell?. Int J of Men's Health.

[55] Larsson C, Ekvall Hansson E, Sundquist K, Jakobsson U (2016). Impact of pain characteristics and fear-avoidance beliefs on physical activity levels among older adults with chronic pain: a population-based, longitudinal study. BMC Geriatr.

[56] Lee J, Kim D, Ryoo H, Shin B (2016). Sustainable wearables: wearable technology for enhancing the quality of human life. Sustainability.

[57] Teixeira E, Fonseca H, Diniz-Sousa F, Veras L, Boppre G, Oliveira J, Pinto D, Alves A, Barbosa A, Mendes R, Marques-Aleixo I (2021). Wearable devices for physical activity and healthcare monitoring in elderly people: a critical review. Geriatrics (Basel).

[58] Gal R, May AM, van Overmeeren EJ, Simons M, Monninkhof EM (2018). The effect of physical activity interventions comprising wearables and smartphone applications on physical activity: a systematic review and meta-analysis. Sports Med Open.

[59] (2020). Caregiver burnout: how technology brings help and hope. HIMSS.

[60] Reinhard S, Given B, Petlick N, Bemis A, Hughes RG (2008). Supporting family caregivers in providing care. Patient Safety and Quality: An Evidence-Based Handbook for Nurses.

[61] Larnyo E, Dai B, Larnyo A, Nutakor JA, Ampon-Wireko S, Nkrumah EN, Appiah R (2022). Impact of actual use behavior of healthcare wearable devices on quality of life: a cross-sectional survey of people with dementia and their caregivers in Ghana. Healthcare (Basel).

[62] Ometov A, Shubina V, Klus L, Skibińska J, Saafi S, Pascacio P, Flueratoru L, Gaibor DQ, Chukhno N, Chukhno O, Ali A, Channa A, Svertoka E, Qaim WB, Casanova-Marqués R, Holcer S, Torres-Sospedra J, Casteleyn S, Ruggeri G, Araniti G, Burget R, Hosek J, Lohan ES (2021). A survey on wearable technology: history, state-of-the-art and current challenges. Comput Netw.

[63] Henriksen A, Haugen Mikalsen M, Woldaregay AZ, Muzny M, Hartvigsen G, Hopstock LA, Grimsgaard S (2018). Using fitness trackers and smartwatches to measure physical activity in research: analysis of consumer wrist-worn wearables. J Med Internet Res.

[64] Rahimi N, Jetter A (2015). Explaining health technology adoption: past, present, future. Proceedings of the Portland International Conference on Management of Engineering and Technology (PICMET).

[65] Finney Rutten LJ, Davis T, Beckjord EB, Blake K, Moser RP, Hesse BW (2012). Picking up the pace: changes in method and frame for the health information national trends survey (2011-2014). J Health Commun.

[66] (2021). The endless possibilities of wearable technology in healthcare. HIMSS.

